# Pandemetrics: systematically assessing, monitoring, and controlling the evolution of a pandemic

**DOI:** 10.1007/s11135-022-01424-7

**Published:** 2022-06-08

**Authors:** Stefano Barone, Alexander Chakhunashvili

**Affiliations:** 1 Department of Agricultural, Forest and Food Sciences, University of Palermo, Palermo, Italy; 2 Department of Quality and Patient Safety, Karolinska University Hospital, Stockholm, Sweden

**Keywords:** Covid-19, Pandemic, Statistical process control, Data monitoring, Early warning, Statistical surveillance

## Abstract

The still ongoing pandemic of SARS-CoV-2 virus and COVID-19 disease, affecting the population worldwide, has demonstrated the need of more accurate methodologies for assessing, monitoring, and controlling an outbreak of such devastating proportions. Authoritative attempts have been made in traditional fields of medicine (epidemiology, virology, infectiology) to address these shortcomings, mainly by relying on mathematical and statistical modeling. However, here, we propose approaching the methodological work from a different, and to some extent alternative, standpoint. Applied systematically, the concepts and tools of statistical engineering and quality management, developed not only in healthcare settings, but also in other scientific contexts, can be very useful in assessing, monitoring, and controlling pandemic events. We propose a methodology based on a set of tools and techniques, formulas, graphs, and tables to support the decision-making concerning the management of a pandemic like COVID-19. This methodological body is hereby named Pandemetrics. This name intends to emphasize the peculiarity of our approach to measuring, and graphically presenting the unique context of the COVID-19 pandemic.

## Introduction

The fast outbreak of the COVID-19 epidemic due to the SARS-Cov-2 virus has prompted an urgent need for methodological innovation, despite the current systems of disease surveillance in place (Bock et al. 2008; Hisada et al. 2020; Kulessa et al. 2019; Mehl et al. 2020; Zhao et al. 2020). Countries and governments worldwide have responded to the devastating waves of infections and deaths, making considerable efforts to counteract the spread of the epidemic.

However, were these efforts effective everywhere? Did they lead to the desired results? Or is it necessary to shift our efforts and develop something completely new and different? This is what the entire humanity, not only the scientific community, is wondering about (Developing Infectious Disease Surveillance Systems 2020).

Epidemiology is the branch of medicine mostly devoted to the study of epidemics and it makes extensive use of statistical methods. Epidemiological studies for COVID-19 disease started to be promptly published last year (Ibrahim 2020; Laxminarayan et al. 2020; Setel et al. 2020). Statistical surveillance is a field of research where specific methods are developed to support the different aspects of the monitoring and controlling of epidemics. More recently, also computer science has provided support, vitality, and innovation to the field (Amit et al. 2020; Shachar et al. 2020). The need for syndromic surveillance, i.e. the early detection of disease outbreaks, by using state-of-the-art statistical methods and informatics was already noted in the past (Tsui et al. 2008).

In the context of the currently ongoing pandemic of COVID-19, Barone et al. (2020) started developing a set of tools, mostly indices and graphics, in the area of descriptive statistics, to fill the existing gap and support the urgent need for understanding the ongoing phenomenon and its implications. The tools proved to be effective so that a pilot dashboard was also developed and set up online (Demetrix SRL). Here, we find it necessary to further deepen some aspects of a methodology aimed at managing the ongoing pandemic.

By *Pandemetrics,* it is hereby meant a reasoned set of measures related to data collected to monitor a pandemic. It will result in a pandemic dashboard where interested stakeholders can gain new insights on the ongoing phenomena and take actions if and when the need arises. In this work, Pandemetrics mostly refers to the country-level unit of analysis. However, the methods are built in such a way that makes it possible to utilize a region-level, city-level, or any other finer-level unit of analysis.

The article is structured as follows: Sect. 2 provides a brief review of the existing work on dashboard development, with related improvements and refinements; Sect. 3 introduces an early warning method, based on statistical process control concepts and control chart schemes; Sect. 4 presents a set of criticality indexes, showing how it is possible to examine a global situation from different angles; Sect. 5 is devoted to the analysis of mortality data from several perspectives; Sect. 6 provides final remarks with a digression on what can be called pandemic management, i.e. the organizational aspects put in place by different countries to manage the pandemic.

## COVID-19 pandemic dashboard: state-of-the-art

**Fig. 1:**
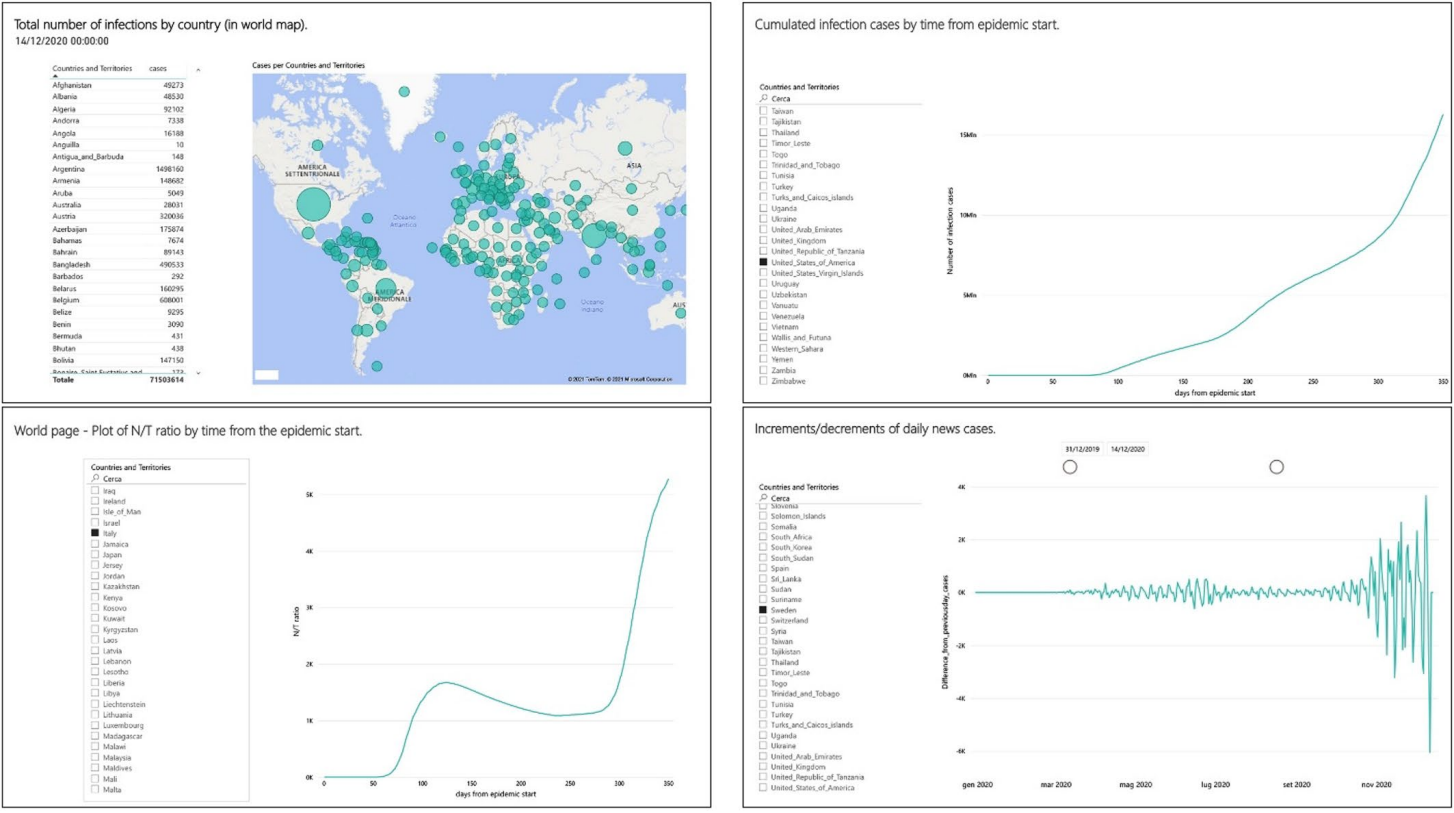
Screenshots of the COVID-19 online technical dashboard

During the first pandemic wave, in March–April 2020, given the need for complete and rigorous dashboards, a descriptive statistical analysis was conducted to better assess the state of the global situation (Barone et al. 2020). Using the data published by the European Centre for Disease Prevention and Control (ECDC) website, they performed a set of exploratory analyses and set up a technical dashboard online, with free public access (Demetrix SRL). Figure 1 shows a collection of screenshots of the online dashboard.

Obviously, that initiative was not isolated. In addition to well-known online dashboards, like John Hopkins (Ncov – JHU CSSE), WHO (WHO Coronavirus (COVID-19) Dashboard), New York Times (Coronavirus World Map: Tracking the Global Outbreak—The New York Times), some others were developed (COVID-19 Data Tracker | IHI—Institute for Healthcare Improvement; COVID-19 in Canada). Most of the efforts to build online dashboards to monitor pandemic evolution were made in the area of informatics engineering (Wissel et al. 2020; Raju et al. 2020; Gong et al. 2020). Pöhler et al. (2021) summarize dashboard development efforts.

## Early warning scheme for pandemic process control

The exploratory data analysis of earlier publications highlighted the need to set up an effective monitoring and control scheme aimed at promptly detecting trends and deviations of the pandemic process in an undesired direction. It should serve as an *early warning* system, which alerts decision-makers to take immediate countermeasures to stop, or at least slow down, the spread of the virus.

An early warning scheme can be grounded upon the concept of statistical process control (SPC) and suitable control charts to timely detect the presence of unwanted variation (Shewhart 1931). There is a wide variety of control charts applicable to different types of processes (Montgomery 2019) and considerable literature on the use of SPC in healthcare research and management (Woodall 2006).

The most critical and sensitive measure of the ongoing pandemic process is related to the variability (increments/decrements) of daily new infection cases. The daily cases are reported constantly by all countries and territories. Within countries, they might be reported by different regions and areas, up to any desired level of analysis.

In SPC, one way to monitor single observations is provided by the Individual—Moving Range charting scheme (henceforth I-MR) (Amin and Ethridge 1998), which consists of two control charts, one for the individual observations and the other for the moving range (difference between the current observation and the previous one).

This scheme is well applicable to the context of COVID-19. To illustrate how the early warning scheme works, we selected six countries representative of very different pandemic evolution. In alphabetical order: Australia, Italy, Singapore, South Korea, Sweden, and the USA.

To use control charts tools, the first step is to define the so-called Phase I, i.e. the phase in which it is possible to assume that the process is stationary (in control) and identify the statistical distribution of the two involved random variables (individual measurement and moving range). Furthermore, we need to identify this phase to define the control charts parameters, central line, and lower/upper control limits. Ideally, a stable condition would be a zero-infections phase, i.e. a pre-pandemic phase. However, it is not possible to set such a condition for identifying Phase I. Thinking pragmatically, Phase I can be considered as a stagnation period in which the spreading of the contagions is very low. All countries have undergone such a phase, most commonly during the summer of 2020.

Given the time series of the individual observations (*n*), defining Phase I needs a pragmatic rule. The rule here adopted is the following:

1. Smooth the time series by a 31-days centered moving average. For each day, the average is calculated by using the earlier 15 days, the current day, and the following 15 days.
2. Find the local minimum in the stagnation phase.
3. Take the 31-day interval around the local minimum as Phase I.

**Fig. 2:**
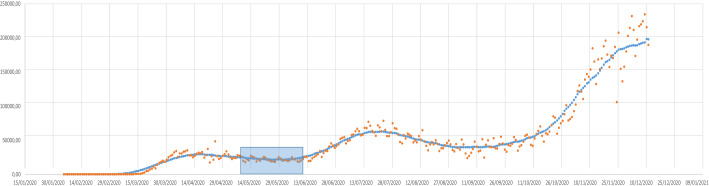
Time series of infection cases for USA and identification of Phase I

Figure 2 shows the time series of the individual observations for the USA, the smoothed time series of the moving average, and highlights Phase I identified by the above-described pragmatic rule.

Once Phase I is identified, it is necessary to study the statistical variability of the individual observations in that “pseudo-stationary” time frame and fit the most suitable random variable (r.v.) model. A first attempt was made to check the fitting of the most plausible model for non-negative integer values, i.e. the Poisson model. Unfortunately, the check was not positive; for all of the six illustrative cases, the variances are much bigger than the averages, which is in contrast with the r.v. model, characterized by a mean value equal to the variance.

**Fig. 3:**
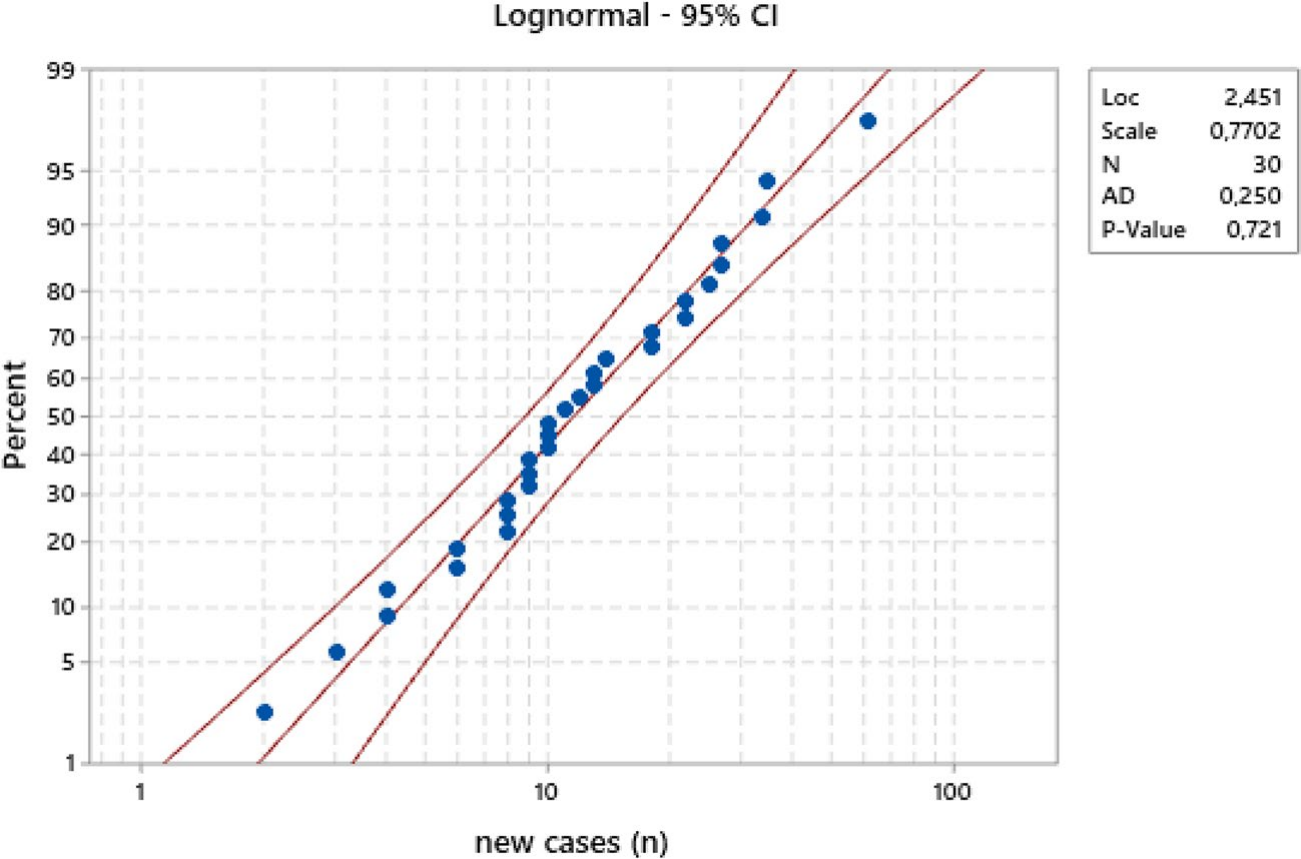
Probability plot for the South Korea subset of new cases in the Phase I timeframe

Therefore, other models were explored and tested including the models of continuous non-negative random variables. The model appearing the most suitable for all six cases is lognormal. Figure 3 shows the probability plot made according to the lognormal model for the subset of South Korea data concerning the daily new cases in Phase I as previously identified (11 April–11 May 2020).

With Phase I identified, and the r.v. model chosen, it is possible to set the control chart parameters. The central line is estimated using the average, and the lower/upper control limits are estimated according to the general rule of “ − / + 3standard_deviation from the average”.

**Fig. 4:**
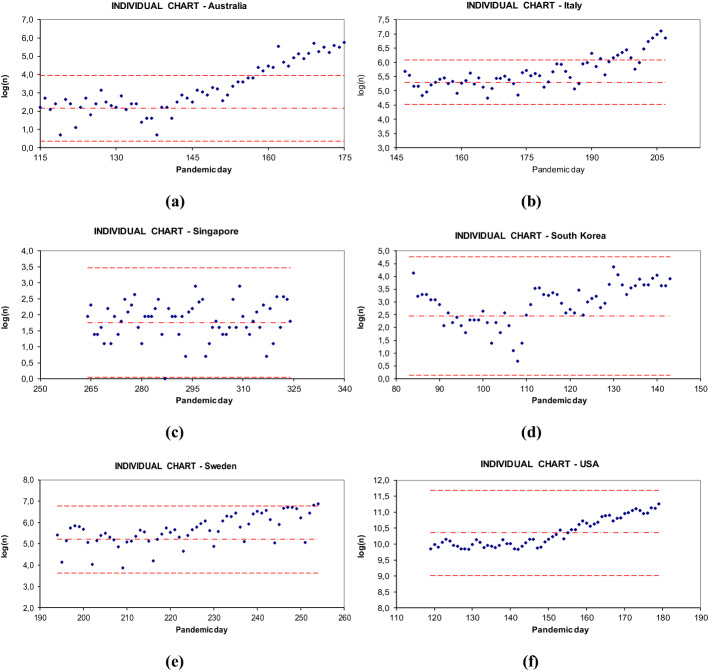
Individual observations control charts for the six selected countries

Figure 4 (panels a through f) shows the individual observation control charts for the six selected countries. The charts plot the data points of Phase I (first 31 points at the left of each chart), plus additional 30 data points subsequent in the time series. The vertical axes report the *log(n)* (assumed to be normally distributed). The horizontal axes report the pandemic days (assuming “pandemic day 1” the first day of a reported case of COVID-19 infection in that country). The pandemic day is used as a time measurement unit to standardize the timeline of the pandemic in different countries. It is possible to observe that Australia and Italy data points go out of control rather soon after the time frame of Phase I, South Korea, Sweden, and the USA show an increasing drift, while Singapore data points keep consistently within control limits. The lower control limit is plotted only for illustration purposes; it is not meant to represent any issue in case a data point falls below that limit.

The individual observations control charts are accompanied by the moving range charts. Preliminary data explorations showed that the best choice is to plot the logarithm of the absolute value of the moving range, *log*(*|MR|*).

The individual observations control chart and the corresponding moving range chart must be plotted and read together, because from the one it is possible to better understand the behavior of the other, and together they provide a complete explanation of the pandemic process evolution.

**Fig. 5:**
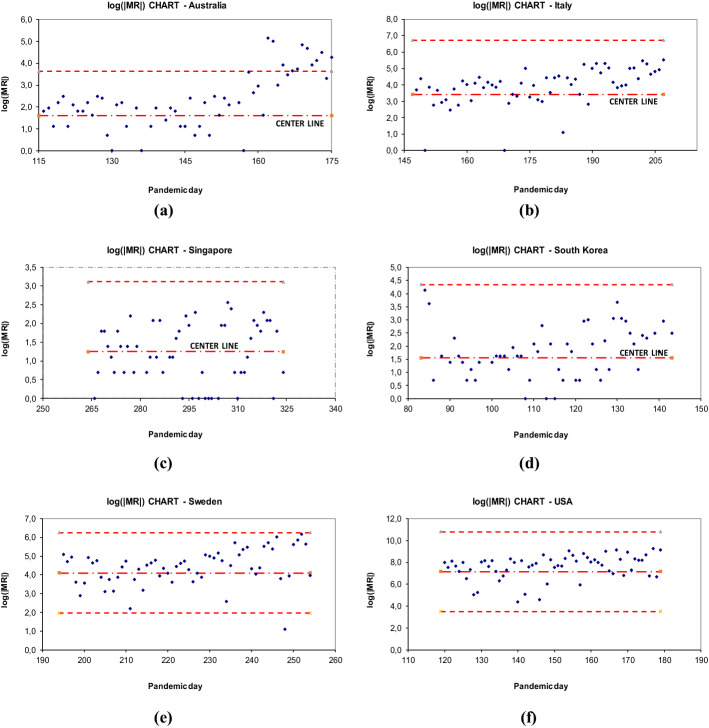
Moving Range control charts for the six selected countries

Figure 5 (panels a through f) shows the control charts of *log(|MR|)* for the six selected countries. As before, the charts represent the data points of Phase I, plus additional 30 data points (days). The vertical axes report the *log(|MR|)*. The horizontal axes report the same time frame of the individual charts in terms of pandemic days. It is possible to observe that the data points of Australia go out of control rather quickly after the Phase I, Italy and Sweden show an increasing drift, while Singapore and USA stay within control limits. Wherever the lower control limit by calculation resulted negative, it was not plotted for obvious reasons.

Naturally, the control charts parameters can be updated, whenever a new stagnation phase happens. For example, in Australia, the period of October-December was a second stagnation period, where the numbers of new cases were even lower than in the period of May–June (first stagnation period). Therefore, an update of Phase I specification is possible and even desirable.

## Criticality analysis

In this Section, a set of criticality indexes is defined, together with a description of their usage. A discussion of the proposed criticality indexes has the objective of showing the strengths and weaknesses of each one, and highlighting new perspectives, to see the evolution of the pandemic and to give the big picture of the phenomenon. The following is based only on a daily updated database. The visuals could be expanded if the database itself is expanded or other more fine-grained data sources at lower levels of analysis are used.

**Fig. 6:**
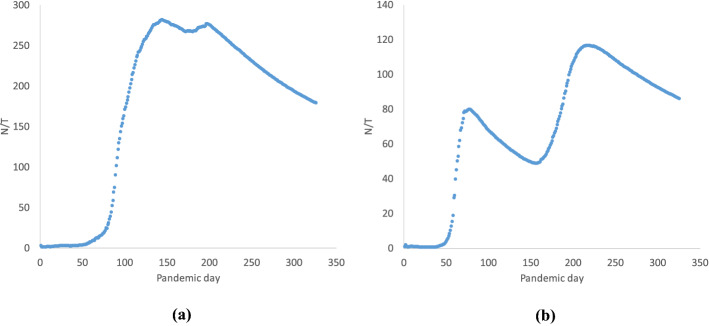
N/T ratio for **a** Singapore, **b** Australia

In Barone et al. (2020) the variable N/T was defined as the ratio between N, representing the cumulative number of cases in a given country and the corresponding pandemic day. In analogy with Physics, this index can be seen as an average pandemic speed, since N can be thought of as the distance covered by the virus in that country, up to the pandemic day T. The average pandemic speed varies over time. It can be increasing, remain stable, or be decreasing. A trend that would be good to observe is an average pandemic speed firstly increasing, then stabilizing, then decreasing towards an asymptotic limit of zero (Fig. 6a Singapore case).

In the case of more than one pandemic wave, it is possible to see a hump trend of the curve (Fig. 6b Australia). In Australia, the trend is such that the second hump is higher than the first one. By looking at these curves, it is easier to observe the real trends of the pandemic in the different countries.

The average pandemic speed is a good indicator, but it is not enough to allow comparisons between countries, which are characterized by very different populations and land areas, and consequently different population densities and distributions. We seek to find an appropriate way to compare the infection numbers of different countries, such as the USA, Sweden, and Italy. These three countries have very different characteristics, first of all in terms of population size (henceforth indicated as **P**) and land area (henceforth indicated as **A**).

**Fig. 7:**
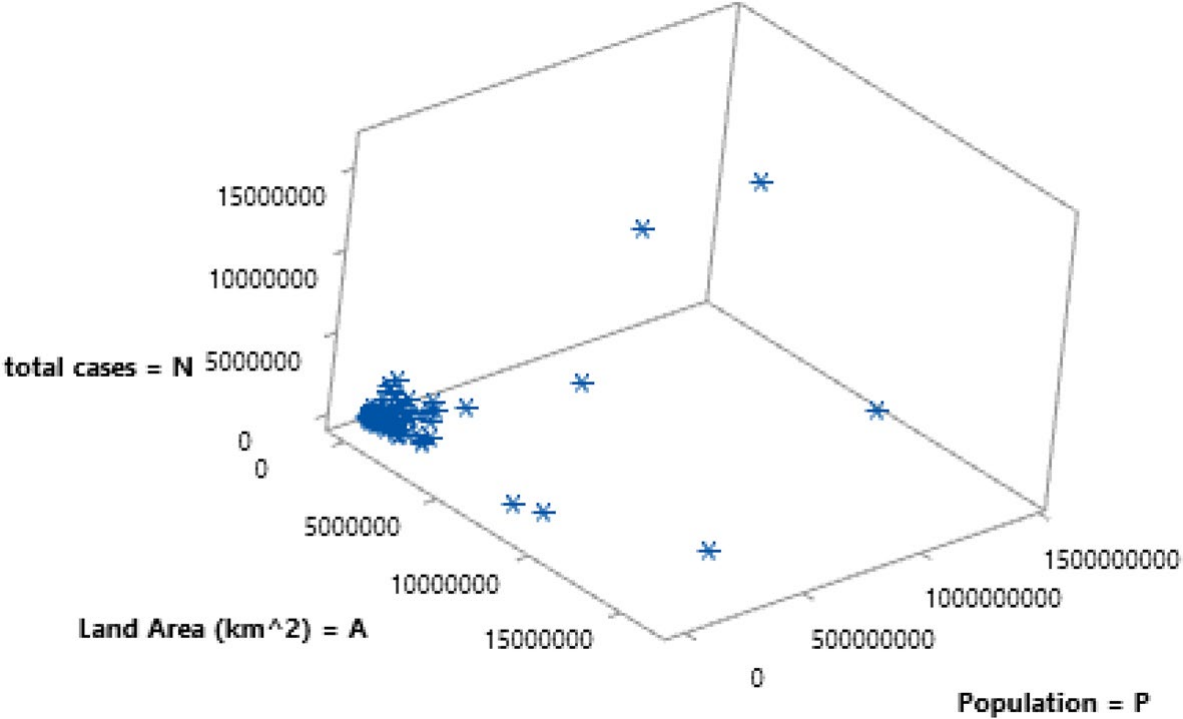
Three-dimensional scatterplot **N**, **P**, **A**

**Fig. 8:**
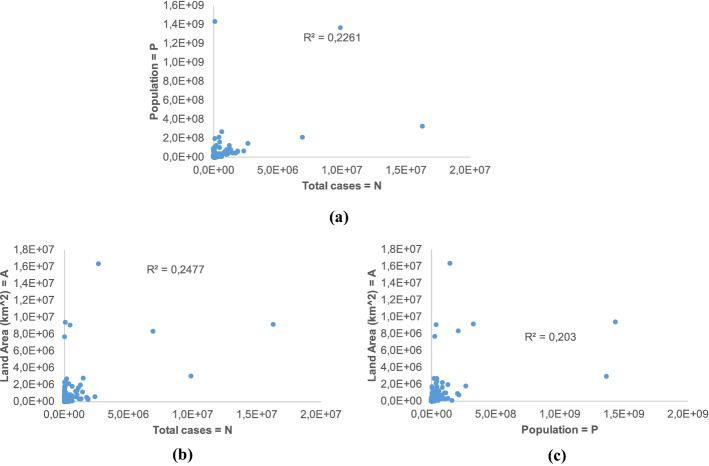
Bivariate scatterplots of the three variables **N**, **P**, and **A**

It is helpful to visualize the tridimensional space of the variables **N**, **P**, and **A** in a 3D scatterplot, where each point represents a country (Fig. 7). The plot is not very clear because there are quite a few outliers, affecting the axes’ scale. However, it seems that there is no special trend; a certain sparsity of the data points can be noted. Projecting the points in two dimensions, three bivariate scatterplots are obtained (Fig. 8).

From the graphs, it is clear the absence of any striking correlation (very low coefficients of determination R^2^ are also shown in each graph). Even between **P** and **A**, the correlation is quite poor, which suggests that there is a large variety of population density between countries.

Having selected these three variables to take into consideration for a criticality analysis, one possible way to harmonize the data comparison between countries was already anticipated in Barone et al. (2020). The basic idea is to divide the total number of cumulative cases, **N**, by the population size, **P**, obtaining the ratio **N/P**. This index can be considered as a sort of *infection rate*. In epidemiology, this ratio is known as prevalence (Ceylan, 2020).

In considering the ratio **N/P** however, there remains a problem of comparing different geographical areas, because having one million people living in a wide geographical area is quite different than having the same million people living in a much smaller area; in other words, population density must be taken into account. Let **A** be the land area of a country (the land area excludes the areas covered by water, like lakes and rivers), the *population density* is computed as **P/A**. Such a ratio can also be considered a criticality index. Furthermore, another possible bivariate combination is the ratio **N/A**. This ratio can be considered as an index of *infection density*.

**Fig. 9:**
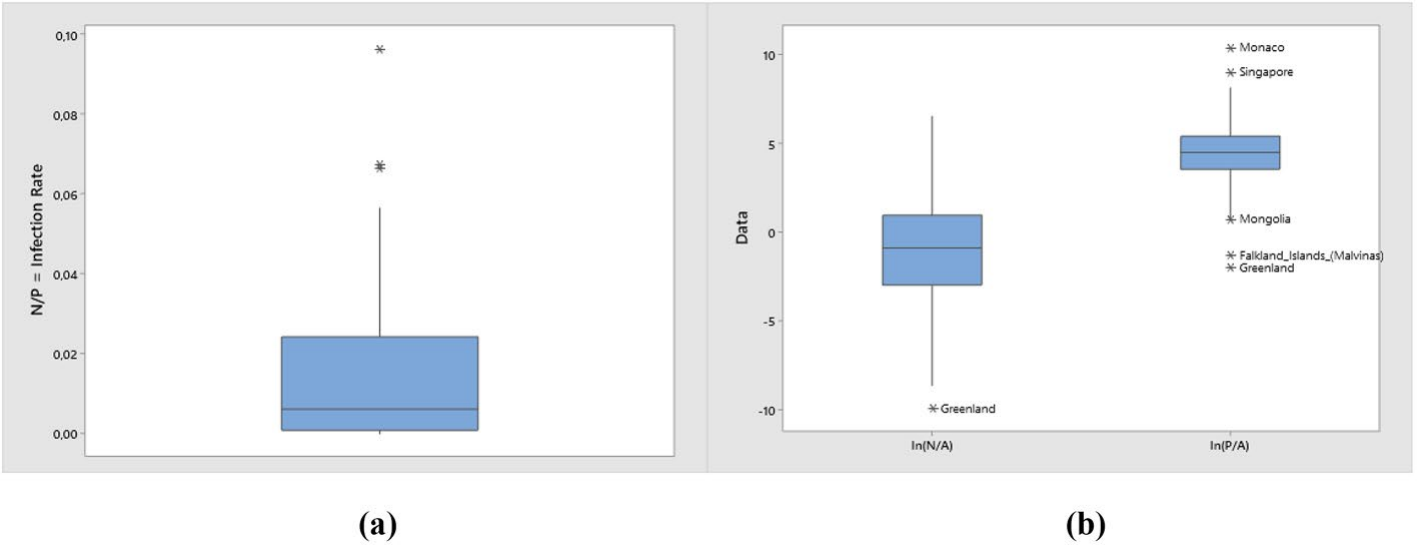
Empirical distributions of the bivariate ratios **N**/**P** (in original scale), **N**/**A**, and **P**/**A** (in logarithmic scale)

The previous three indexes use only two variables at a time, and it is quite evident that a high infection rate **N/P** means high criticality, a high population density **P/A** means high criticality, a high infection density **N/A** means high criticality. Figure 9 shows the empirical distribution of the three bivariate criticality indexes. For **N/A** and **P/A**, a preliminary log transformation was necessary for a smoother visualization on the box-whiskers plot.

Notice that in the above three bivariate indexes, **N** is always in the nominator and **A** always in the denominator, but **P** is once in the nominator and once in the denominator. Thus, the role of **P** is twofold. This is a key issue in defining and utilizing a composite criticality index. When confronted with the number of infections, **P** is better placed in the denominator, meaning that the higher the population to the number of infections, the more relaxed one can be. When confronted with the land area (available space), the higher the population, the worse it is, because people are closer to each other, and this can determine a higher risk of infection spreading.

The three bivariate indexes are an interesting set to monitor. However, let us consider what happens if we try to combine the three variables altogether. The goal here is to define a composite criticality index. The reasoning to formulate a single criticality index is not immediate, because of the role of **P**, which is twofold as mentioned above.

One possible three-variate criticality index can be obtained by combining **N**, **P**, **A,** in what seems to be the most reasonable way. High criticality should relate to high **N**, high **P** (because the higher the population exposed to the infection, the higher the risk), and small **A**. Therefore, the index **NP/A** is defined. Table 1 shows the list of the top 20 Countries sorted by descending **NP/A**. For simplification of table reading, the criticality index NP/A was divided by 10^6^.

**Table 1:** Top twenty Countries in descending order of criticality index **NP**/**A** (divided by 10^6^)

Country	NP/A
India	4500
Bangladesh	610
United States of America	580
United Kingdom	510
Singapore	480
Italy	380
Germany	320
Netherlands	310
France	290
Belgium	230
Bahrain	190
Brazil	170
Philippines	160
Spain	160
Israel	140
Poland	140
Pakistan	120
Turkey	110
Palestine	100
Lebanon	98

Alternative reasoning can be the following: starting from the infection rate **N/P**, we may say that the same infection rate in a geographically wider country is less critical. Thus, another criticality index can be defined as **N/(PA)**. According to this reasoning, even moderate infection rates but in very small areas need to be considered critical. Table 2 presents the list of the top twenty countries in descending order of **N/(PA)**. For simplification of table reading, the criticality index N/(PA) was here multiplied by 10^6^.

**Table 2:** Top twenty countries in descending order of criticality index **N**/(**PA**) (multiplied by 10^6^)

Country	N/(PA)
Monaco	20,000
Gibraltar	3200
San Marino	940
Sint Maarten	850
Aruba	260
Liechtenstein	250
Andorra	200
Bermuda	140
Maldives	84
Guam	79
Bahrain	71
Malta	70
Curaçao	50
United States Virgin Islands	49
Montserrat	26
Luxembourg	26
Turks and Caicos islands	21
Cayman Islands	19
British Virgin Islands	17
French Polynesia	15

From this perspective, the cases of Italy, Sweden, and the USA do not seem to be very critical; while some countries are very critical in both perspectives, like, for example, Bahrain, which ranks in the top 20 in both lists. Both perspectives are arguable, but both look reasonable.

Notice that, despite **P** being in the nominator of the first index and the denominator of the second, the two indexes are not reciprocals. This can be easily seen if we do the ranking firstly by the former and then by the latter. The top Countries in the first ranking are not at the bottom in the second ranking.

A contradiction seems to arise in defining the two three-variate indexes. For a given N and A, the position of P in the numerator of one index and the denominator of the other one results in opposite conclusions regarding the criticality. Is criticality, intended as the severity of the situation of a given country, directly proportional to the amount of population, or inversely proportional to it? The key to untangling this conundrum may lie in adopting and monitoring the two indexes at different stages of the pandemic evolution.

**Fig. 10:**
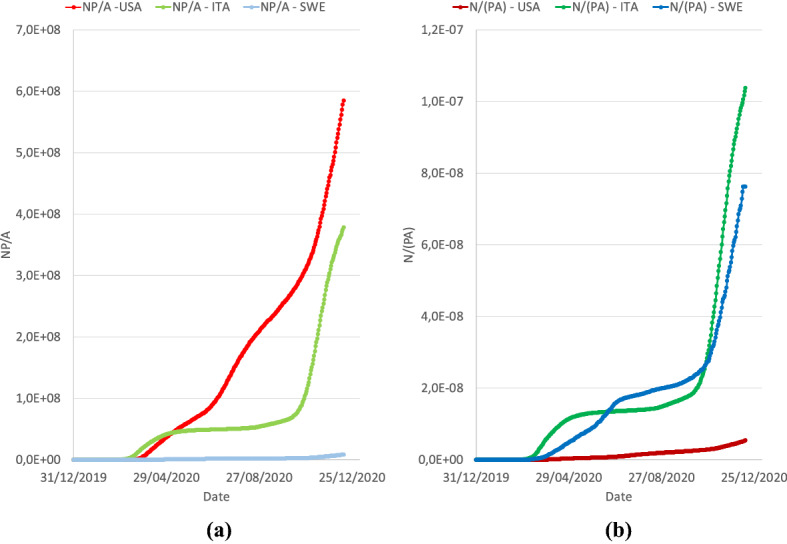
Trends of criticality indexes for USA, Italy, and Sweden. **a** index NP/A; **b N**/(**PA**)

Figure 10 shows the trend of the two indexes vs. time for the three countries the USA, Italy, and Sweden; Fig. 10a concerns the index **NP**/**A**, and Fig. 10b concerns the index **N**/(**PA**).

Notice that in terms of NP/A the criticality of Sweden is always low, while the USA and Italy have values that are dramatically higher exhibiting different trends over time. Conversely, in terms of **N**/(**PA**) it is the USA that shows a low criticality, while Italy and Sweden lie dramatically higher exhibiting similar trends.

Let us recall that the temporal evolution of the values of the above-defined indexes for any given country is only dependent on the number of cumulative cases, N, because P and A are constant (or can be thought as constant, P clearly changes over time). As a result, the usage of these indexes is intended more as a tool for comparison between countries (but also within countries if data at finer levels are available), rather than a monitoring tool inside a given country, because over time they both simply increase.

Further reasoning is needed to resolve the apparent contradiction that the two proposed composite indexes in three variables seem to suggest with respect to the relationship between criticality and population. Let us consider for example two countries of the same land area, A, and with, at a given point in time, the same number of cumulative cases, N. The country with a larger population is the one where the current situation can be considered the most critical, in the sense that more people spread in the same area, are exposed to a certain number of infections, and so are at risk of being infected. This rationale refers to the idea of population density because the index NP/A can be seen as a composite of N and P/A. Here we are using a definition of criticality that relates to the risk of infection, as the potential of having the population exposed to the spread of the virus. This index is then to be monitored during the spread of the pandemic, to give an early warning to the densest countries (or regions, or cities), where the same number of cases has a greater potential of doing more damage than in a sparsely populated area.

However, as the severity of the pandemic was starting to be acknowledged, many countries adopted containment measures to limit the spread of the virus and its consequences. Isolating the infected patients, limiting social interactions, and modifying the daily routines of the people residing in many highly affected areas, have contributed to the slowing down of the spread of the virus.

The index N/(PA) could be useful to compare the efficacy of different measures adopted by different countries. If two countries have the same population and area (so also same population density), a country with a lower value of N/(PA) than the other has proved to manage better the separation between infected and non-infected cases, while the other has allowed more spread of the infection. Given that the two have the same starting conditions of area and population, one has performed better than the other in containment.

Another important thing to consider is that most countries do not have a uniform distribution of their population on their land area. Thus, the values at the aggregate country level may not be as descriptive as desired. For example, Canada has an extensive land area, and the population is very sparsely concentrated. Indeed, by looking at the P/A population density seems very low. However, the reality is that most of the country is not inhabited, and the large city areas are just as dense as any city in the USA. Thus, caution is needed when aggregately comparing countries.

One solution to overcome this issue could be to look at some large cities of comparable size and population, so comparable density, and then evaluate the number of cases in such cities. For example, one could select all metropolises with at least 1 million inhabitants, and compare and contrast the values of the index N/(PA) to see who has dealt with the pandemic better and discuss how they did it. Even more importantly, it could be useful to compare countries effective management by comparing the rate of acceleration of these indexes. 

## Mortality analysis

In this section, we turn the attention towards the reported number of deaths related to COVID-19, as reported by the different countries and daily updated by the ECDC (up to 14 December 2020). We suggest approaches to monitor the mortality related to the spread of the virus being aware of the limitations concerning the data collection and analysis. Indeed, different countries have different protocols in place to test the most severe cases and to report the eventual cause of death of a patient as related to COVID-19 or not.

Finding the true mortality rate of COVID-19 is not a simple question to answer (Chang et al. 2020; Freitas et al. 2020; West et al. 2020; Fricker 2021). If we look at the total number of cases and the total number of deaths across all different countries, we see a huge variation.

**Fig. 11:**
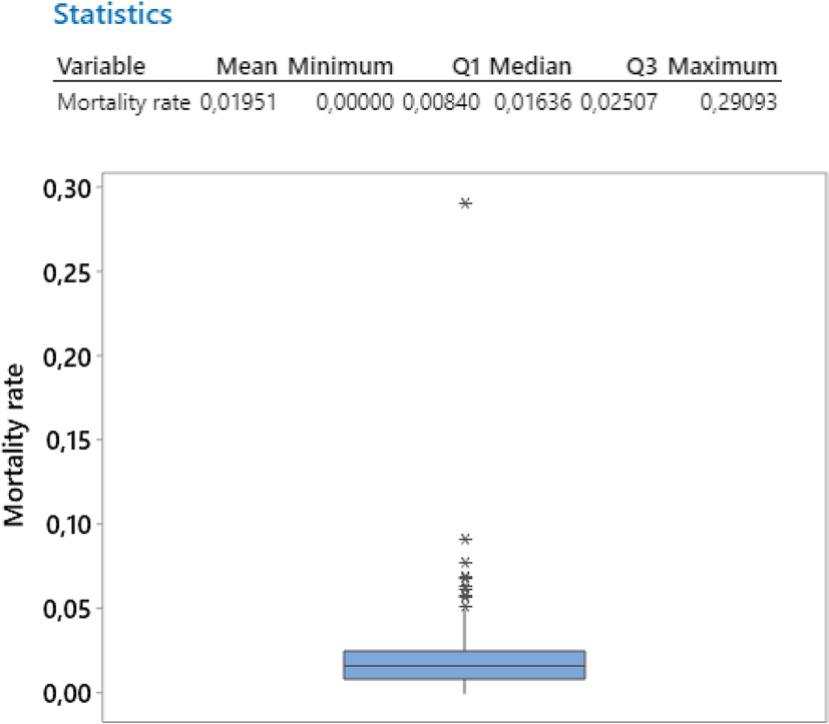
Empirical distribution of the mortality rate (all countries)

Consider the definition of the mortality rate of D/N, where D and N are computed based on the same date (again subscripts indicating time and country are omitted here for simplicity). The empirical distribution of the mortality rate shows a certain variation (Fig. 11). However, there are many outliers, Yemen being the maximum with an incredible 29% mortality rate.

In analyzing the mortality rate, a large variation may be explained by the different testing policies introduced and implemented by the countries (affecting the denominator of how many cases are recorded) as well as by the different rules/regulations to attribute a death to COVID-19. The borderline between the “death caused by COVID-19” and the “death with COVID-19” is not always clear, which in turn causes a variation in the way countries are reporting the new cases of death.

**Fig. 12:**
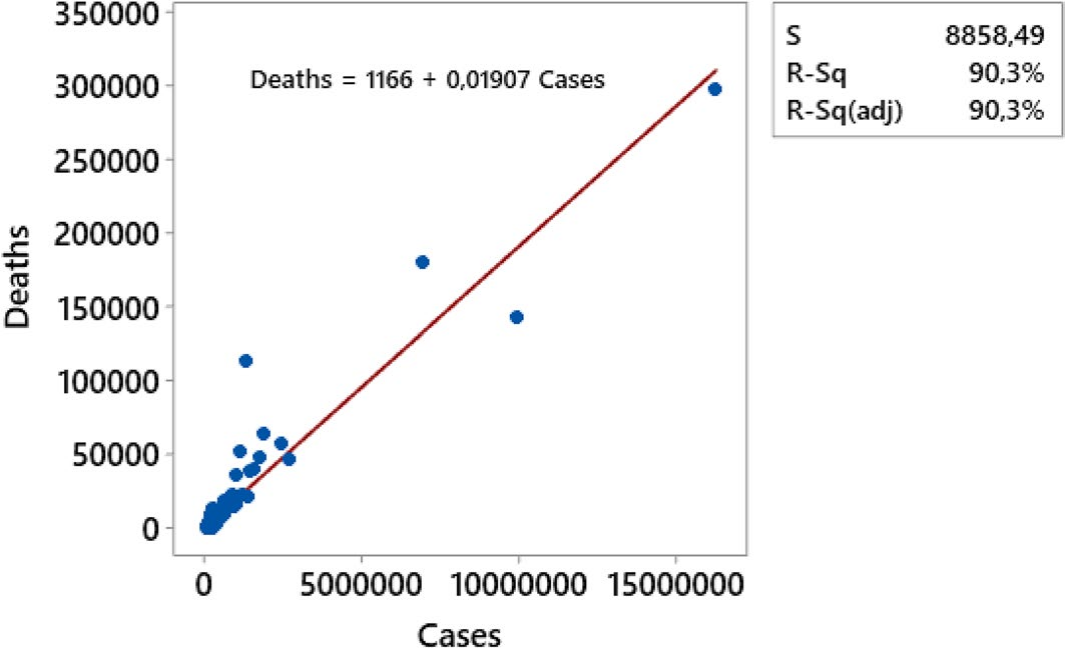
Scatterplot cumulated deaths D vs. cumulated infection cases N

As intuition suggests, the number of deaths is highly correlated with the number of cases. The scatterplot of cumulated deaths vs cumulated cases for different countries, as illustrated in Fig. 12, shows a rather linear behavior (R^2^ = 90.3%).

Exceptionally outstanding cases are Mexico (1,250,044 infections vs 113,953 deaths), Brazil (6,901,952 infections vs 181,402 deaths). Conversely, despite a large number of cases, India seems to be a singular case of low mortality rate (9,884,100 infections vs 143,355 deaths).

**Fig. 13:**
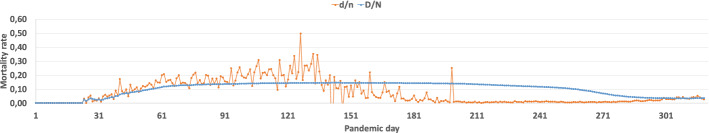
Cumulative and daily mortality rate in Italy

Furthermore, together with the cumulated mortality rate, citizens are used to seeing reports of daily counts of new cases and new deaths. So, another kind of proxy for mortality rate is provided as a general perception given by d/n, where d is the daily number of deaths and n the daily number of new cases. Such measure, even if not explicitly reported by the media, often creates the perception of a daily mortality rate that can be quite deceiving. One reason is certainly that there is a lag effect between the number of cases and the number of deaths, having the deaths of any given day likely been counted as new cases some days or weeks before. This aspect will be analyzed later in this section. Moreover, it is a quite variable measure that also depends on the updating of such numbers; thus, for example, the numbers often decline over the weekends because they are later reported at the beginning of the following week (let us recall that the primary work of the doctors is to cure and save the lives of their patients, not to keep the numbers continuously updated). Figure 13 shows for the case of Italy the striking variability of the daily mortality rate (red line), especially when compared to the trend of the cumulative mortality (blue line).

The cumulative mortality rate is simply computed as D/N at any given point in time. The reason to look at the cumulative mortality rate is twofold. On the one hand, it avoids the short-sided, and deceiving perception of the daily ratios of new deaths over new cases, which is the daily mortality measure; on the other hand, it helps to show the trend of how the deadly effects of the virus are evolving. This helps to compare different countries, and in the long run will inform on the true mortality rate of the virus itself, disentangled from the measurement differences that characterize the ways different countries count both the cases and the deaths. Moreover, it can highlight how the countries are fighting the virus and improving in taking care of the most severely sick patients.

**Fig. 14:**
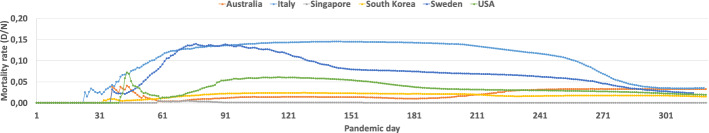
Cumulative mortality rate in the six selected countries

Figure 14 shows the cumulative mortality rate for the six selected countries. The cumulative mortality rate reported here starts from pandemic day 1 (the day of the first reported case in a given country, so they may be different calendar days for different countries). The graph shows that there is an initial spike that tends to decline over time in different ways for different countries.

It is quite striking to see the differences between the peaks of these curves for the different countries. The European countries, such as, Italy and Sweden suffered higher peaks of cumulative mortality rate, at least assuming the uniformity of the reported numbers across countries. Italy kept a high mortality rate for a long time.

Another interesting finding, evident from these graphs, is that all curves seem to slowly converge in the long run towards a common value of mortality rate, which is reasonable to expect.

### The time lag between cases and deaths

**Fig. 15:**
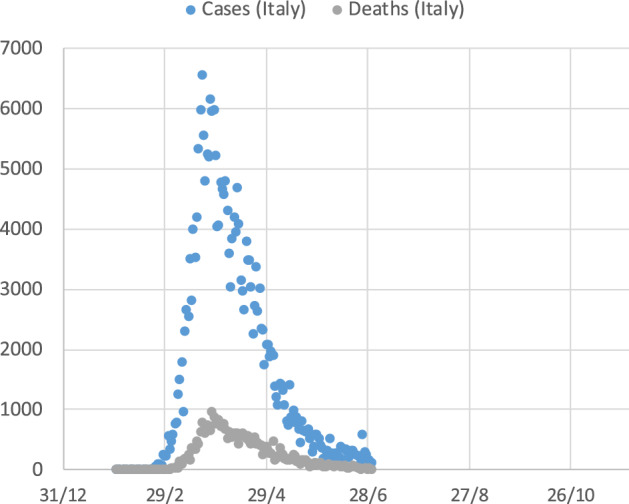
Daily infection cases and deaths in Italy during the first pandemic wave

One of the interesting aspects in analyzing mortality data is to understand the relationship between the two variables of the number of cases, **n**, and the number of deaths, **d**. Examining the scatter plots of daily cases and deaths vs time, the relationship between the two series is evident in several countries. Figure 15 shows the plot of the time series of new cases and deaths, daily reported in Italy in the first pandemic wave between January and June 2020.

**Fig. 16:**
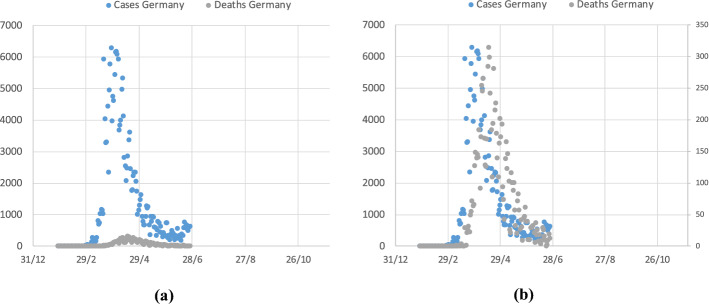
Daily infection cases and deaths in Germany during the first pandemic wave. **a** same vertical axis scale, **b** independent vertical axis scale

The correlation between the two series is evident. For other countries, it is not so evident, but this can be simply due to the different order of magnitude of the two series; a problem which can be solved by plotting the two series on the same graph but with two different vertical axes. Figure 16 shows the illustrative case of Germany. When adopting the two separate scales, the two series visibly match, showing only a time lag of some days, since deaths, in most cases, occur some days after the reported infection.

We can examine the relationship between cases and deaths to extrapolate information about the time lag effect. For each country, the simplest statistical model can be generally written as:

$$  d_{{t_{i} }} = \beta_{0} + \beta_{1} \cdot n_{{t_{i - l} }} + \epsilon $$

where the subscript refers to the time of observation, *l* is the time lag, $\beta_{0}$ and $\beta_{1}$ are the intercept and slope of the linear regression model, $\epsilon$ is the error term.

To determine the lag effect, the above linear regression model can be fitted for several choices of *l*, then the optimal model will be the one with the maximum explained variance. For example, by fitting the model to Italy data (first pandemic wave, considering a period from 31/01/2020 to 30/06/2020), we find that the optimal value is 7. Therefore, the best predictor for the number of deaths is the number of cases recorded seven days before. The first pandemic wave in Italy was in fact very dramatic probably because healthcare was unprepared to face the disease and people were not adequately cared for at their homes. Moreover, the registration of the positive testing was done only at hospital admission when probably was too late.

The time lag differs from country to country (e.g., it is estimated in 10 days in Australia and 8 days in the USA in the first pandemic wave), the reasons may be due to different care protocols, different population demographics, different climate, and so on, in addition to and the above-mentioned different testing and reporting policies.

Such a model is useful for a first-order interpretation, but it may be too simple to employ for predictive purposes. Indeed, there is no magic number that always predicts how many days a patient survives the virus, and there is substantial variation between patient groups and patient characteristics.

One way to increase the predictive value of these models is to add more predictors such as more lagged variables of number of cases, more demographic predictors, or construct a more comprehensive regression model that will lead to a higher R-square and predictive value; however, such model may lose interpretability.

### Mortality in different pandemic waves

The pandemic has so far developed in waves. Every country has so far undergone two or three waves. This is a well-known phenomenon in epidemiology, characterizing also previous epidemics. We noticed that some countries had a higher mortality ratio in the second pandemic wave than in the first wave: Australia, Iran, Israel for example. In other countries instead, the mortality ratio was lower in the second wave e.g., Ireland, Japan, USA.

It seems there is no “statistical law" governing this aspect of the pandemic evolution.

### Additional information on COVID-19 death counts

As said before, there are different rules to the attribution of a death to COVID-19. However, another source of information can be based on the analysis of the difference between official death counts data (all deaths reported in a country) of the year 2020 and the previous years. A previous study on this line of thought was done by (Freitas et al. 2020). For example, for November 2020 in Italy were officially reported 76,291 deaths (official data source the Italian Statistical Institute, ISTAT), the average number of deaths in November in the previous five years (2015–2019) was 51,462 (source: Italian Statistical Institute, ISTAT). Thus, the difference of deaths between November 2020 and November previous five years is 24,829, but only 16,583 deaths were officially reported as due to COVID-19 (source ECDC). Hence, it is hard to say what justifies such a big difference of 8,246 deaths. One possibility is that the official number of deaths is underreported; another possible explanation is that the excess deaths are a collateral effect of the pandemic, and they are resulting from difficulties in care for unrelated causes of death (people don’t seek care for fear of contracting the virus, or hospitals have congested departments and the whole health care system suffers directly and indirectly).

## Conclusions: pandemic management

The analysis of the COVID-19 pandemic with the methods presented in this article gives the possibility to discuss several managerial aspects of the pandemics. The evidence that emerged from the different perspectives indicated that a pandemic is akin to a real war that needs a special organization, strategy and discipline. Some countries are winning the war, by keeping the spreading of contagions low and consequently have a low number of deaths.

The analysis of the trends with simple graphics for easy reading and interpretation, put together in a clear dashboard (such as the one discussed in Sect. 2) helps decision-makers, but also ordinary readers to obtain a full picture of the ongoing phenomenon. However, this is not enough.

The Early Warning SPC scheme here presented (in Sect. 3) is an important method to carefully monitor the pandemic process in any populated place, it can be used at any level of community aggregation (municipality, region, state, and so on) and it is a way to get an immediate alert of increasing danger so that it is possible to take the adequate countermeasures and bring back the situation under control.

The criticality indexes here presented (in Sect. 4) provide numerical tools to effectively show the current criticality status of a place and can be used to easily compare several places in a single table. They can be updated automatically daily, as far as daily data are reported and published.

The analysis of mortality (in Sect. 5) shows a strong correlation between contagions spread and deaths as well as it illustrates a time lag effect between infection reported and death. Despite quite a big variation between countries, these phenomena follow clear statistical laws. Moreover, data show a big mismatch between official death counts and what is likely to be the real numbers. Therefore, there is much to do to rigorously classify a death as a direct consequence of the COVID-19 disease.

In addition, there is a problem in counting the number of daily new cases. These data can be affected by many sources of confounding factors, e.g., different testing policies, absence of any sampling strategy for testing, and so on. The accuracy of testing itself can be affected by false positive and false negative, and the latter is very detrimental for population safety (West et al. 2020).

Analyzing all these aspects in summary we can say that the countries winning the pandemic war are mostly countries of Asia (e.g., China, South Korea, Singapore) and Oceania (e.g., Australia), probably due to their experience on previous epidemics—in the case of Asia (Kim 2020), and higher isolation and possibility to better control their boundaries, in the case of Oceania.

In summary, we can say that the strategy to adopt should consist of keeping low the contagions, in three main ways:

- Mass screening. Basically, everybody must be compelled to undergo a reliable diagnostic test, for free. No charges. All people must be screened, as soon as possible. If testing the whole population is not affordable for any reason at a certain time, then it is necessary to adopt correct sampling techniques. There is a wide literature on statistical sampling, but so far there are no occurrences of such approach in combatting the COVID-19 epidemic.
- Massive high-tech contact tracing. Basically, everybody having a cellphone must be compelled to install and run a contact tracing app. The life of just one person is much more important than the privacy of thousands of people. Privacy concern is a false problem, firstly because people movement data are recorded through our smartphones and saved (a user can check his/her movements up to years before) unless explicitly interdicted by the user through some hidden option; secondly because by letting someone know that he/she was in close contact with anybody else who resulted positive to covid-19 testing is just an additional information which can be very useful for the recipient, who can immediately check him/herself and/or putting him/herself on quarantine. Only by a massive use of contact tracing apps, it is possible to get an effective result in terms of virus spreading reduction.
- Strict quarantine for infected and “potentially infected” people, then one can quit the quarantine only if he/she tests negative to the virus. Strict quarantine should be systematically applied to people entering the borders of the country.

These are the three basic recommendations that countries should seriously follow.

Countries following these prescriptions are living normal lives inside their boundaries. Countries that are not following these prescriptions are wasting more resources (for healthcare and economic support), create more trouble to citizens (freedom restrictions for total or partial lockdowns), and register more victims.

In addition to the previous three general prescriptions, it seems that an enlarged and intelligent use of information technology and communications without big concerns (in most cases not founded) on privacy limitations may have saved millions of human lives (Bae et al. 2020; Jia and Yang, 2020).

This work has some limitations, e.g., for a complete criticality analysis there might be other variables in addition to N, P, and A to be possibly taken into account. This work does not discuss the healthcare pathways and processes, which clearly have a huge importance on the containment of the pandemic. These aspects were intentionally kept out of the scope of this work, which is mostly related to higher-level data management issues. For those interested, an updated review work is provided in Pietrantonio et al. (2021).
